# Supplementary material for: How conspicuous are peacock eyespots and other colorful feathers in the eyes of mammalian predators?
Source: PLoS One. 2019 Apr 24;14(4):e0210924. doi: 10.1371/journal.pone.0210924 (PMC6481771; doi:10.1371/journal.pone.0210924)
Supplement: S1 Appendix — (DOCX) [file pone.0210924.s001.docx]

**S1 Appendix. Predation on wild Indian peafowl**

| **Predator species (adult peafowl)** | **Reference** |
| --- | --- |
| Adult male peafowl found to be 0.8% of kills recorded for dholes (*Cuon alpinus*), leopards (*Panthera pardus*) and tigers (*Panthera tigris*), far less than the 9-10% of prey recorded in the study area in India | [1] |
| Evidence that leopards prey on peafowl in the study area in India at levels less than their availability | [2] |
| Reported low occurrence of peafowl remains in leopard scats (-4%) in the study area in India. | [3] |
| Reported low occurrence of peafowl remains in leopard scats (2.9%), and none for dholes or hyenas in India | [4] |
| Peafowl present in study area in India, and reported to be potential prey of leopards and tigers, but no evidence found of predation in scat. | [5] |
| Peafowl reported to be potential prey of tigers, leopards and dholes in study area in India, but no evidence for predation found by scat analysis or remains of kills | [6] |
| No evidence found for predation on peafowl by tigers in India from scat analysis. | [7] |
| Peafowl not mentioned in review of prey selection by leopards world-wide | [8] |
| Peafowl described as a significantly avoided prey species for tigers in a review of prey selection by tigers world-wide. | [9] |
| Predation on peafowl reported to take place primarily in the first year based on a review of the literature; two fatalities (one adult male, one adult female) reported for the study area and period. | [10] |
| Wild dogs, jungle cats (*Felis chans*), foxes, jackals (*Canis aureus*), and snakes listed as predators on adult peafowl in India | [11] |
| Tigers listed as predator on adult peafowl in India | [12] |
| Wild and domestic dogs (*Canis lupus familiaris*) listed as predators on adult peafowl in India | [13,14] |
| Asiatic lion (*Panthera leo persica*), leopard, jackal, jungle cat and crested hawk eagle (*Spizcietus cirrhatus*) listed as predators on adult peafowl in India | [15] |
| Adult peafowl reported to be preyed on by leopards, lions and jackals in India | [16] |

**References**

1. Johnsingh AJT. Large mammalian prey-predators in Bandipur. Journal of the Bombay Natural History Society. 1983;80: 1–57.

2. Mondal K, Gupta S, Qureshi Q, Sankar K. Prey selection and food habits of leopard (Panthera pardus fusca) in Sariska Tiger Reserve, Rajasthan, India. mammalia. 2011;75: 201–205. doi:10.1515/mamm.2011.011

3. Sankar K, Johnsingh AJT. Food habits of tiger (Panthera tigris) and leopard (Panthera pardus) in Sariska Tiger Reserve, Rajasthan, India, as shown by scat analysis. Mammalia. 2002;66: 285–288.

4. Arivazhagan C, Arumugam R, Thiyagesan K. Food habits of leopard (Panthera pardus fusca), dhole (Cuon alpinus) and striped hyena (Hyaena hyaena) in a tropical dry thorn forest of southern India. Journal of the Bombay Natural History Society. 2007;104: 178–187.

5. Ramesh T, Snehalatha V, Sankar K, Qureshi Q. Food habits and prey selection of tiger and leopard in Mudumalai Tiger Reserve, Tamil Nadu, India. Journal of Scientific Transactions in Environment and Technovation. 2009;2: 170–181.

6. Kumaraguru A, Saravanamuthu R, Brinda K, Asokan S. Prey preference of large carnivores in Anamalai Tiger Reserve, India. European Journal of Wildlife Research. 2011;57: 627–637.

7. Biswas S, Sankar K. Prey abundance and food habit of tigers (Panthera tigris tigris) in Pench National Park, Madhya Pradesh, India. Journal of Zoology. 2002;256: 411–420.

8. Hayward MW, Henschel P, O’Brien J, Hofmeyr M, Balme G, Kerley GIH. Prey preferences of the leopard (Panthera pardus). Journal of Zoology. 2006;270: 298–313. doi:10.1111/j.1469-7998.2006.00139.x

9. Hayward MW, Jędrzejewski W, Jêdrzejewska B. Prey preferences of the tiger Panthera tigris. Journal of Zoology. 2012;286: 221–231. doi:10.1111/j.1469-7998.2011.00871.x

10. de Silva PK, Santiapillai C, Dissanayake S. Some aspects of the population ecology of the blue peafowl, Pavo cristatus, in Ruhuna National Park, Sri Lanka. Journal of South Asian Natural History. 1996;2: 113–126.

11. Ashok M, Chaturvedi S. Threats, and Conservation of Indian Peafowl Pavo Cristatus in Bharatpur, Rajasthan: Present Status. International Journal of Pharmacology and Biological Sciences; Jalgaon. 2016;10: 1–8.

12. Harihar A, Pandav B, Goyal SP. Responses of tiger (Panthera tigris) and their prey to removal of anthropogenic influences in Rajaji National Park, India. Eur J Wildl Res. 2009;55: 97–105. doi:10.1007/s10344-008-0219-2

13. Johnsingh AJT, Murali S. The ecology and behaviour of the Indian peafowl (Pavo cristatus) Linn. of Injar. J Bombay Nat Hist Soc. 1978;75: 1069–1079.

14. Rajeshkumar N, Balasubramanian P. Habitat use and food habits: of Indian peafowl (Pavo cristatus) in Anaikatty Hills, Western Ghats. Indian Birds. 2011;7: 125–127.

15. Trivedi P, Johnsingh AJT. Roost selection by Indian Peafowl (Pavo cristatus) in Gir Forest, India. Journal of the Bombay Natural Histroy Society. 1996;93: 25–29.

16. Dookia S. Ecology and Behaviour of Indian Peafowl (Pavo cristatus) in Keoladeo National Park, Bharatpur, Rajasthan, India. International Journal of Fauna and Biological Studies. 2015;2: 97–103.
