# Supplementary material for: How conspicuous are peacock eyespots and other colorful feathers in the eyes of mammalian predators?
Source: PLoS One. 2019 Apr 24;14(4):e0210924. doi: 10.1371/journal.pone.0210924 (PMC6481771; doi:10.1371/journal.pone.0210924)
Supplement: S2 Appendix — (DOCX) [file pone.0210924.s002.docx]

**S2 Appendix. Sources of images used for textural analysis of photographs of live Indian peacock trains photographed in native habitats**

| A | Athula Edirisinghe, ML140316741, Bundala National Park (General), Southern Province, Sri Lanka | Macaulay Library at the Cornell Lab of Ornithology |
| --- | --- | --- |
| B | Joanna Watson, ML141692751, Yala National Park (General), Southern Province, Sri Lanka | Macaulay Library at the Cornell Lab of Ornithology |
| C | Ragoo Rao, IBC980486. Photo of Indian Peafowl *Pavo cristatus* at Mysore District, India. Accessible at [hbw.com/ibc/980486](https://www.hbw.com/ibc/980486). | Internet Bird Collection |
| D | Martin Flack, IBC1052473. Photo of Indian Peafowl *Pavo cristatus* at Jim Corbett National Park, India. Accessible at [hbw.com/ibc/1052473](https://www.hbw.com/ibc/1052473). | Internet Bird Collection |
| E | Shantilal Varu, IBC1263156. Photo of Indian Peafowl *Pavo cristatus* at Bhavnagar District, India. Accessible at [hbw.com/ibc/1263156](https://www.hbw.com/ibc/1263156) | Internet Bird Collection |
| F | Andrew Emmerson, IBC1105223. Photo of Indian Peafowl *Pavo cristatus* at Yala (Ruhunu) National Park, Sri Lanka. Accessible at [hbw.com/ibc/1105223](https://www.hbw.com/ibc/1105223). | Internet Bird Collection |
| G | Andrew Emmerson, IBC1106045. Photo of Indian Peafowl *Pavo cristatus* at Yala (Ruhunu) National Park, Sri Lanka. Accessible at [hbw.com/ibc/1106045](https://www.hbw.com/ibc/1106045). | Internet Bird Collection |
| H | Jaysukh Parekh Suman, IBC1521786. Photo of Indian Peafowl Pavo cristatus at Kutch District, India. Accessible at [hbw.com/ibc/1521786](https://www.hbw.com/ibc/1521786). | Internet Bird Collection |
| I | Nimali Digo and Thilanka Edirisinghe, IBC1280192. Photo of Indian Peafowl *Pavo cristatus* at Embilipitiya, Sri Lanka. Accessible at [hbw.com/ibc/1280192](https://www.hbw.com/ibc/1280192) | Internet Bird Collection |
| J | Nikhil Adhikary, IBC1106912. Photo of Indian Peafowl *Pavo cristatus* at Jalpaiguri District, India. Accessible at [hbw.com/ibc/1106912](https://www.hbw.com/ibc/1106912) | Internet Bird Collection |
| K | Athula Edirisinghe, IBC1058481. Photo of Indian Peafowl *Pavo cristatus* at Medirigiriya, Sri Lanka. Accessible at [hbw.com/ibc/1058481](https://www.hbw.com/ibc/1058481) | Internet Bird Collection |
| L | Ragoo Rao, IBC965853. Photo of Indian Peafowl *Pavo cristatus* at Mysore District, India. Accessible at [hbw.com/ibc/965853](https://www.hbw.com/ibc/965853). | Internet Bird Collection |
| M | Shantilal Varu, IBC984780. Photo of Indian Peafowl *Pavo cristatus* at Kutch District, India. Accessible at [hbw.com/ibc/984780](https://www.hbw.com/ibc/984780) | Internet Bird Collection |
| N | Shantilal Varu, IBC1270807. Photo of Indian Peafowl *Pavo cristatus* at Gir National Park & Lion Sanctuary, India. Accessible at [hbw.com/ibc/1270807](https://www.hbw.com/ibc/1270807). | Internet Bird Collection |
