# Supplementary material for: How conspicuous are peacock eyespots and other colorful feathers in the eyes of mammalian predators?
Source: PLoS One. 2019 Apr 24;14(4):e0210924. doi: 10.1371/journal.pone.0210924 (PMC6481771; doi:10.1371/journal.pone.0210924)
Supplement: S3 Appendix — (DOCX) [file pone.0210924.s003.docx]

**S3 Appendix. Species of plants used for background foliage in multispectral images.**

To determine the mixture of color, brightness and textures of green vegetation to use as background for the multispectral images, we consulted photographs at the Macauley Library (Cornell University), the Internet Bird Collection [1] and Fig S2b in [2], as well as [3–7]. Parrot feather samples were mounted on a background of a freshly-cut saucer magnolia (*Magnolia x soulangeana*) leaves for multispectral imaging, and the same species was used as for comparison with peacock eyespots and blue peacock plumage. The various green leaves analyzed for comparison with the various feather samples are shown in S3 Fig. In the model train photographs, the tree species comprised littleleaf linden (*Tilia cordata*), black locust (*Robinia pseudoacacia*), and elm (*Ulmus americana*), while the brush was a mixture of *Phytolacca decandra*, *Cirsium arvense*, *Helianthus divaricatus*, *Arctium*, *Eupatorium maculatum*, and *Persicaria perfoliata*. The grass species were a mix of *Poa pratensis* and *Lolium perenne*.

**References**

1. Lynx Promocions, S.L. Photos of Indian Peafowl (Pavo Cristatus). In: The Internet Bird Collection [Internet]. [cited 25 Feb 2019]. Available: https://www.hbw.com/ibc/species/53521/photos?title=indian%20peafowl&uid=&sort_by=value&sort_order=DESC&items_per_page=20&page=1

2. Yorzinski JL, Patricelli GL, Babcock JS, Pearson JM, Platt ML. Through their eyes: selective attention in peahens during courtship. Journal of Experimental Biology. 2013;216: 3035–3046. doi:10.1242/jeb.087338

3. Chopra G, Kumar T. A Study of Food and Feeding Habits of Blue Peafowl, Pavo Cristatus Linnaeus, 1758 in District Kurukshetra, Haryana (India).

4. Johnsingh AJT, Murali S. The ecology and behaviour of the Indian peafowl (Pavo cristatus) Linn. of Injar. J Bombay Nat Hist Soc. 1980;75: 1069–1079.

5. Kushwaha S, Kumar A. A review on Indian peafowl (Pavo cristatus) Linnaeus (1758). J Wildl Res. 2016;4: 42–59.

6. Rajeshkumar N, Balasubramanian P. Habitat use and food habits: of Indian peafowl (Pavo cristatus) in Anaikatty Hills, Western Ghats. Indian Birds. 2011;7: 125–127.

7. de Silva PK, Santiapillai C, Dissanayake S. Some aspects of the population ecology of the blue peafowl, Pavo cristatus, in Ruhuna National Park, Sri Lanka. Journal of South Asian Natural History. 1996;2: 113–126.
