# Supplementary material for: How conspicuous are peacock eyespots and other colorful feathers in the eyes of mammalian predators?
Source: PLoS One. 2019 Apr 24;14(4):e0210924. doi: 10.1371/journal.pone.0210924 (PMC6481771; doi:10.1371/journal.pone.0210924)
Supplement: S4 Appendix — (DOCX) [file pone.0210924.s004.docx]

**S4 Appendix. Methods for spatially filtering multispectral images to account for visual acuity effects at varying viewing distances.**

Before spatial filtering of multispectral images, viewing distance, D, and object dimensions on the image, X, in pixels were converted into effective viewing angle in the observer’s visual field using θ = atan(X/D) × 180 deg/π. Blurring due to spatial filtering by the eye’s point spread function was modeled using a Gaussian blur filter (MATLAB *imgaussfilt)* with sigma = (2.9 x visual acuity)^-1^ [1]. For peafowl, we used a visual acuity = 20.6 cycles/deg calculated from retinal anatomy [2]. To model predator and parrot vision, visual acuity was set to 8 cycles/deg, in agreement with values measured for various parrot species [3], the Eurasian lynx, a wild cat similar in size to leopards [4], jackals and dholes [5], and domestic cats, domestic dogs, wolves and foxes [6]. The visual acuity for domestic cats under scotopic conditions was found to be reduced [7,8], with 4 cycles/deg reported in [7]. The effect of the camera optics was determined by measuring the minimum resolvable angle [9], determined by measuring the smallest line-pair spacing resolvable on images of a spatial resolution test standard photographed at 20 cm (0.410$\pm$0.005 mm) and 2.0 m (6.32 $\pm$ 0.02 mm); this set a minimum effective viewing distance.

These calculations also showed that discretization artefacts noted in [10] were minimal because the peafowl and predator visual acuity disks correspond to large numbers of pixels on the multispectral images (44 and 255 pixel^2^ at 2 m; 60,000 and 950 pixel^2^ at 20 cm camera-sample distances, respectively).

**References**

1. Raffel M, Willert CE, Scarano F, Kähler CJ, Wereley ST, Kompenhans J. Particle image velocimetry: a practical guide. Berlin: Springer-Verlag; 2018.

2. Hart NS. Vision in the peafowl (Aves: Pavo cristatus). Journal of Experimental Biology. 2002;205: 3925–3935.

3. Caves EM, Brandley NC, Johnsen S. Visual Acuity and the Evolution of Signals. Trends in Ecology & Evolution. 2018;33: 358–372. doi:10.1016/j.tree.2018.03.001

4. Maffei L, Fiorentini A, Bisti S. The visual acuity of the Lynx. Vision Research. 1990;30: 527–528. doi:10.1016/0042-6989(90)90064-R

5. Sillero-Zubiri C, Hoffmann M, Macdonald DW. Canids: foxes, wolves, jackals, and dogs: status survey and conservation action plan. IUCN Gland, Switzerland; 2004.

6. Malkemper EP, Peichl L. Retinal photoreceptor and ganglion cell types and topographies in the red fox (Vulpes vulpes) and Arctic fox (Vulpes lagopus). Journal of Comparative Neurology. 2018;526: 2078–2098. doi:10.1002/cne.24493

7. Kang I, Reem RE, Kaczmarowski AL, Malpeli JG. Contrast Sensitivity of Cats and Humans in Scotopic and Mesopic Conditions. Journal of Neurophysiology. 2009;102: 831–840. doi:10.1152/jn.90641.2008

8. Pasternak T, Merigan WH. The luminance dependence of spatial vision in the cat. Vision Research. 1981;21: 1333–1339. doi:10.1016/0042-6989(81)90240-6

9. Caves EM, Johnsen S. AcuityView: An r package for portraying the effects of visual acuity on scenes observed by an animal. Methods in Ecology and Evolution. 2018;9: 793–797. doi:10.1111/2041-210X.12911

10. Stevens M, Párraga CA, Cuthill IC, Partridge JC, Troscianko TS. Using digital photography to study animal coloration. Biol J Linn Soc. 2007;90: 211–237. doi:10.1111/j.1095-8312.2007.00725.x
