# Supplementary material for: How conspicuous are peacock eyespots and other colorful feathers in the eyes of mammalian predators?
Source: PLoS One. 2019 Apr 24;14(4):e0210924. doi: 10.1371/journal.pone.0210924 (PMC6481771; doi:10.1371/journal.pone.0210924)
Supplement: S1 Fig — (DOCX) [file pone.0210924.s005.docx]

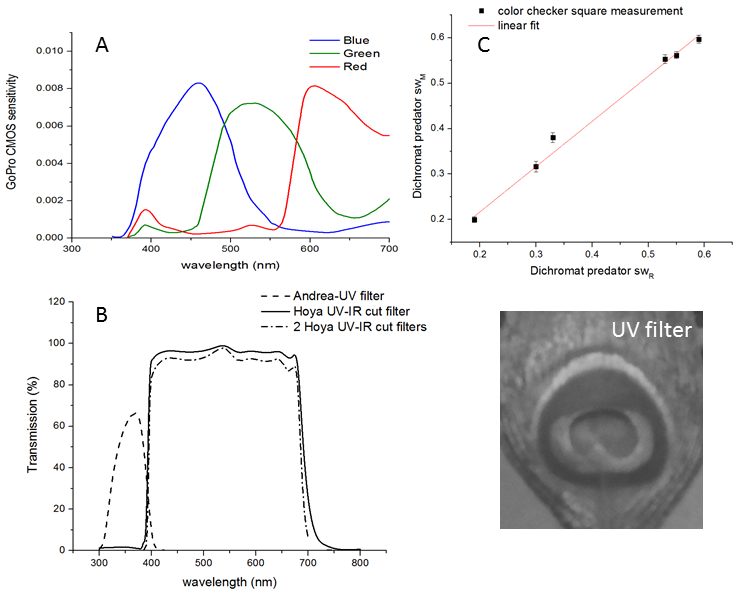


**S1 Fig. Multispectral camera specifications: image sensor spectral response and filter transmission spectra.** (A) Spectral response of the red, green and blue CMOS image sensors in the GoPro Hero 4 camera used for multispectral imaging (normalized to unit area.) (B) Transmission spectra of the Hoya ultraviolet-infrared (UV-IR) cut and Andrea-UV ultraviolet-pass filters used in multispectral imaging. Note that a combination of two of the UV-IR cut filters gives superior IR rejection. Inset: Photograph of an Indian peacock eyespot feather using a full-spectrum modified GoPro Hero 4 Silver Edition camera with the Andrea-UV filter. (C) Validation of the cone mapping model that converts multispectral image measurements into quantum cone catch is shown by the linear relationship with slope one and zero intercept between the dichromatic predator color space coordinate sw values for the color checker chart squares computed from reflectance spectra (x-axis, sw_R_) and from multispectral image data using the MATLAB code (y-axis, sw_M_).
