# Supplementary material for: How conspicuous are peacock eyespots and other colorful feathers in the eyes of mammalian predators?
Source: PLoS One. 2019 Apr 24;14(4):e0210924. doi: 10.1371/journal.pone.0210924 (PMC6481771; doi:10.1371/journal.pone.0210924)
Supplement: S2 Fig — (DOCX) [file pone.0210924.s006.docx]

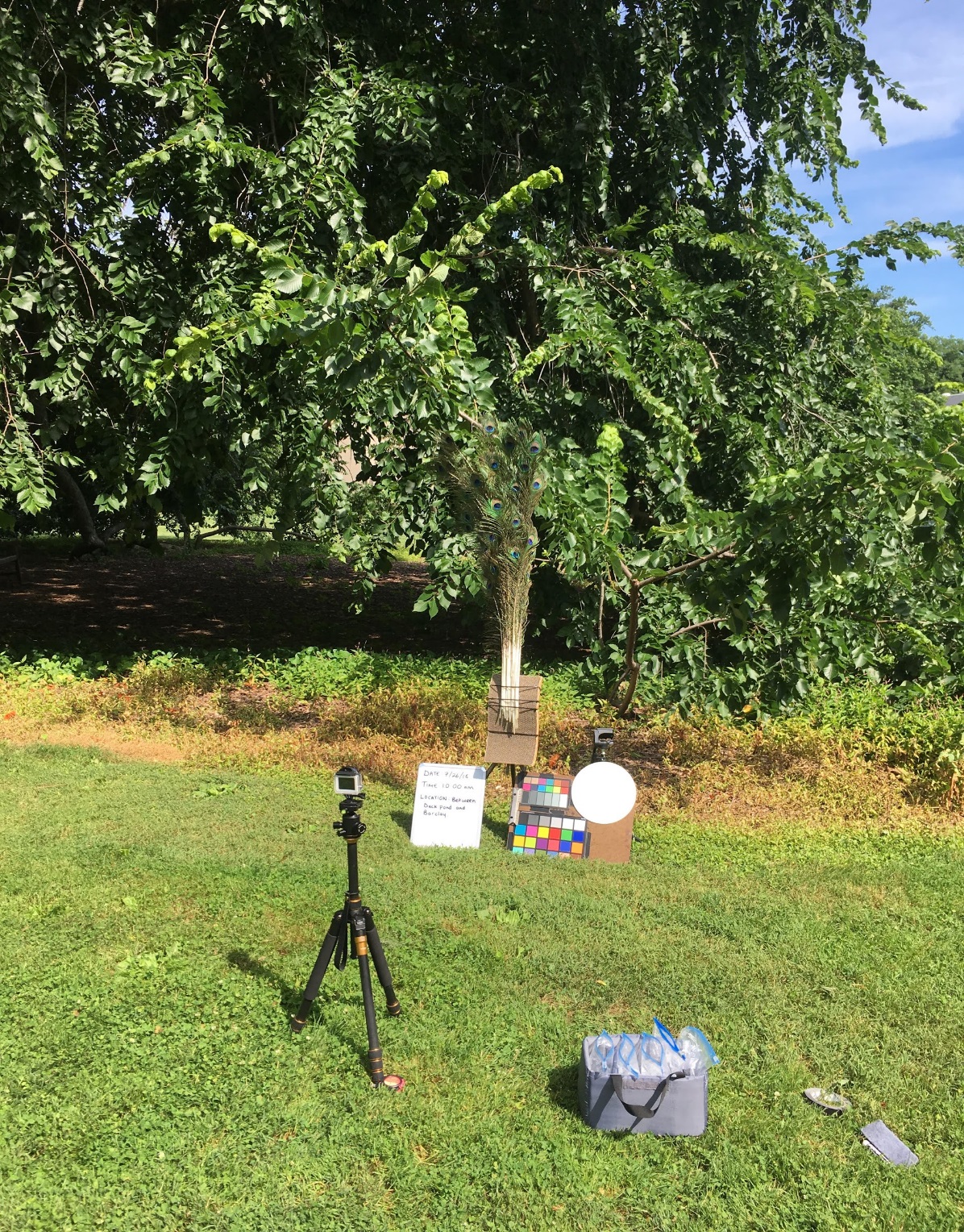

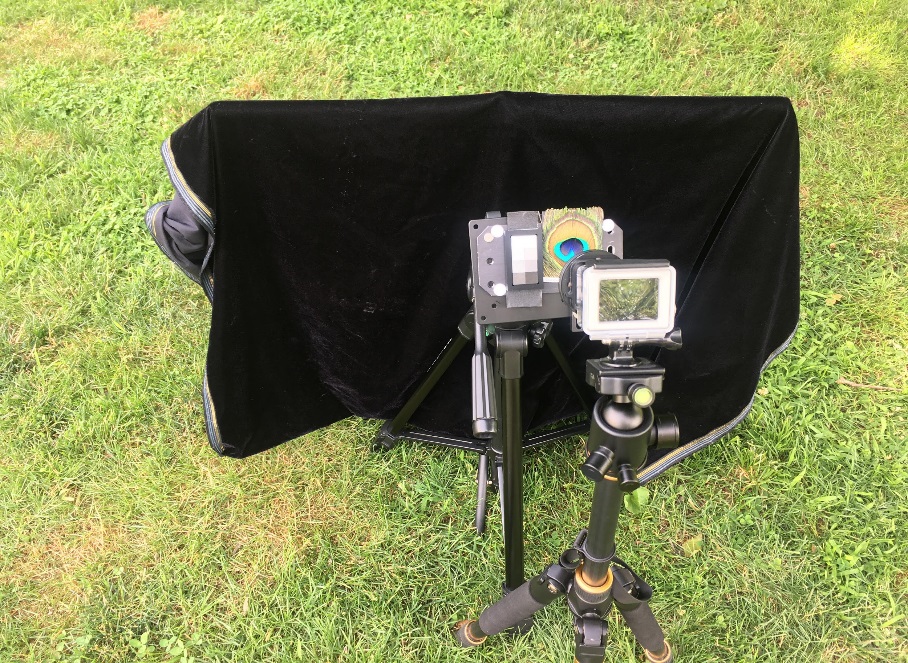


A

C


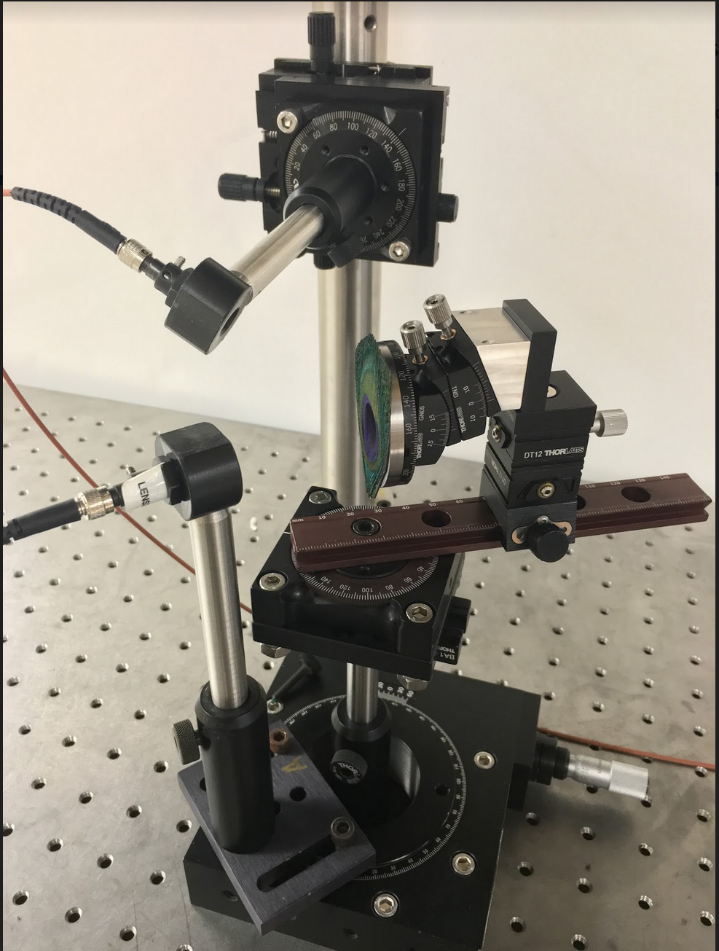


B

**S2 Fig. Reflectance spectroscopy apparatus and multispectral camera filming setup.** (A) Feather reflectance spectroscopy apparatus. Multispectral imaging apparatus for feather samples (B) and the model train (C).
