# Supplementary material for: How conspicuous are peacock eyespots and other colorful feathers in the eyes of mammalian predators?
Source: PLoS One. 2019 Apr 24;14(4):e0210924. doi: 10.1371/journal.pone.0210924 (PMC6481771; doi:10.1371/journal.pone.0210924)
Supplement: S3 Fig — (DOCX) [file pone.0210924.s007.docx]

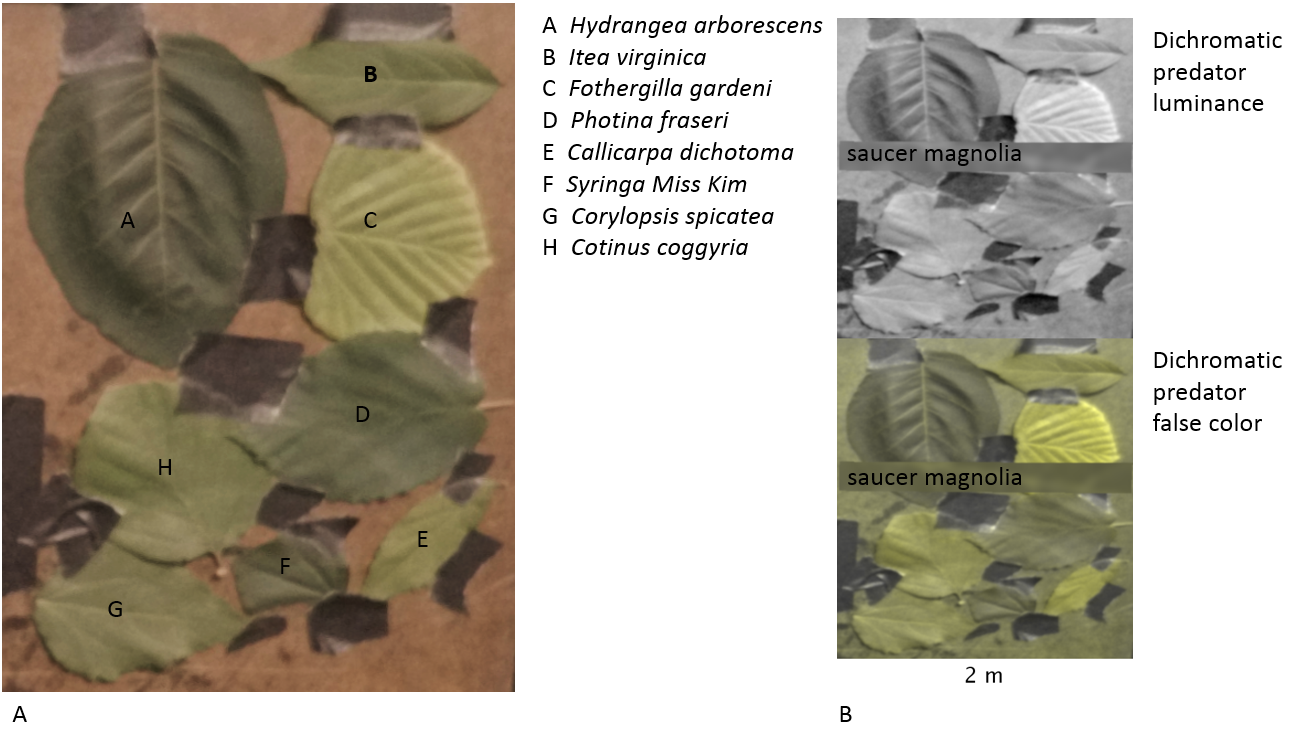


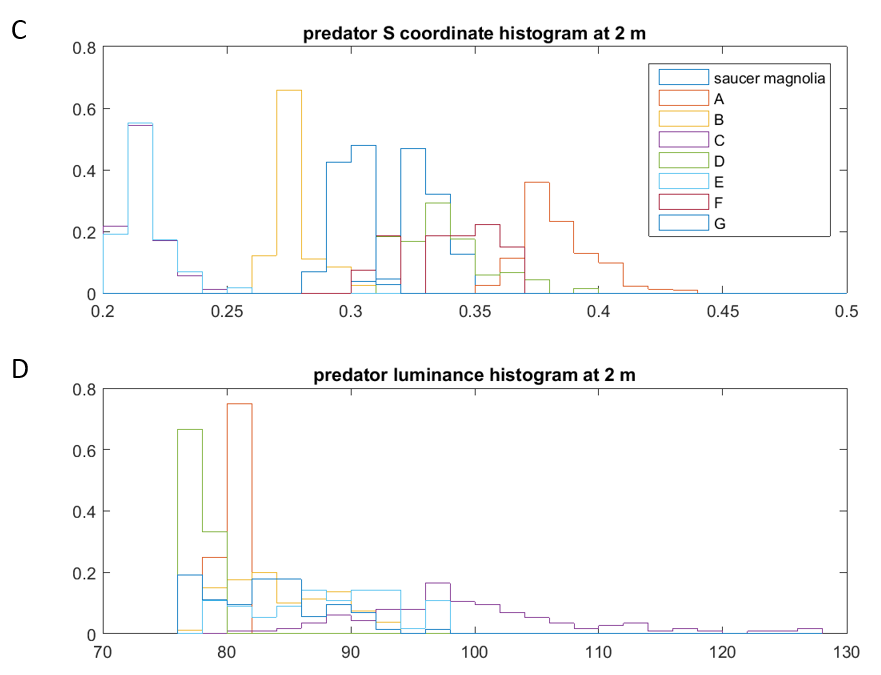


**S3 Fig. Various green leaves imaged and analyzed for comparison with feather samples and the saucer magnolia leaves used as a background.** (A) RGB and (B) dichromatic mammalian predator luminance and false color images. Dichromatic mammalian predator data comparing the leaves shown above with the saucer magnolia leaves used as feather background: (C) S colorspace coordinate and (D) luminance data.
