# Supplementary material for: How conspicuous are peacock eyespots and other colorful feathers in the eyes of mammalian predators?
Source: PLoS One. 2019 Apr 24;14(4):e0210924. doi: 10.1371/journal.pone.0210924 (PMC6481771; doi:10.1371/journal.pone.0210924)
Supplement: S4 Fig — (DOCX) [file pone.0210924.s008.docx]

**
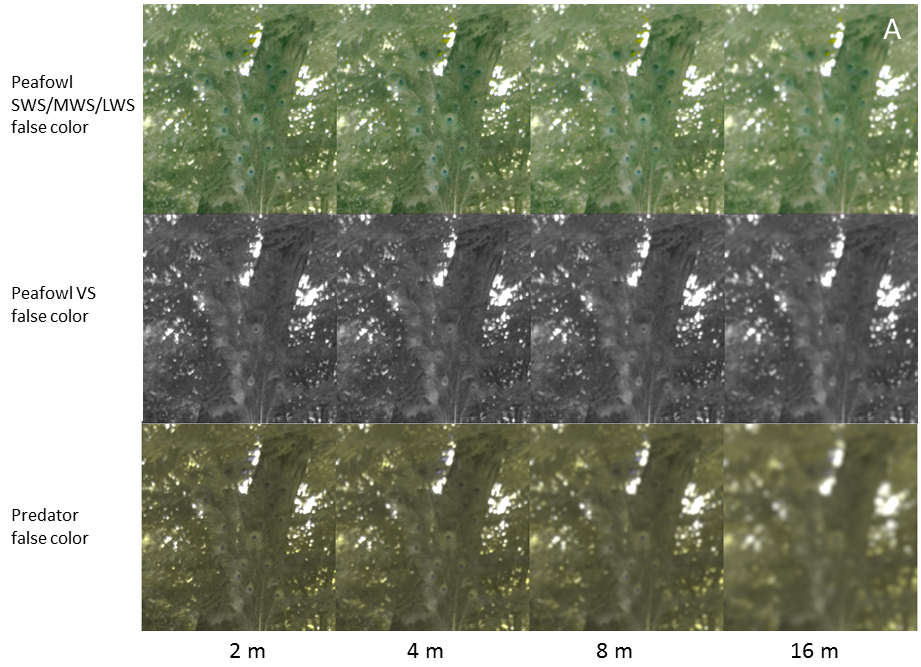
**

**S4 Fig A (caption on next page)**

**
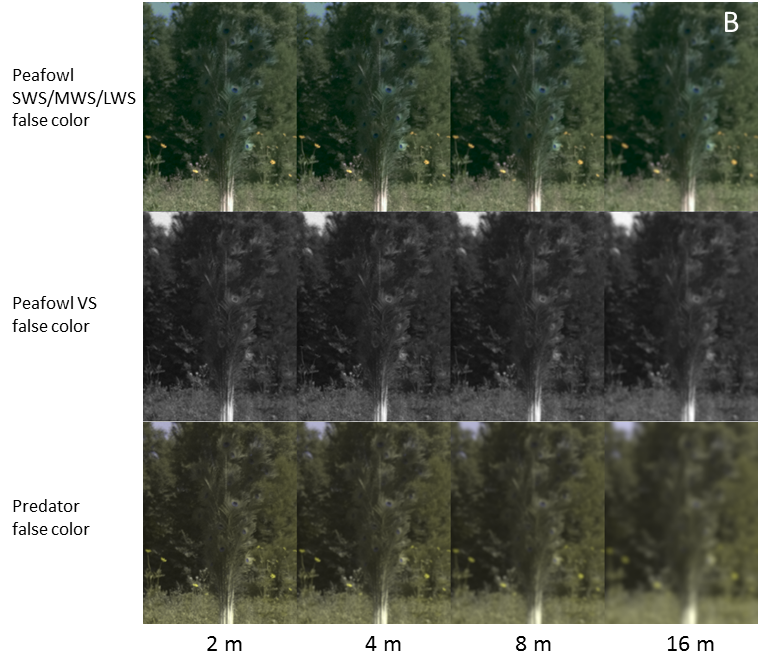
**

**S4 Fig B (caption on next page)**

**
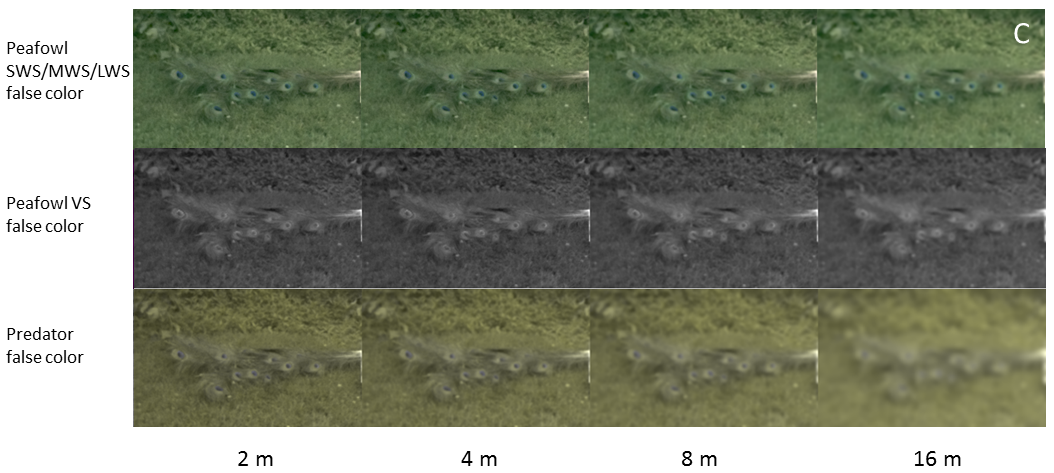
**

**S4 Fig C (caption on next page)**

**
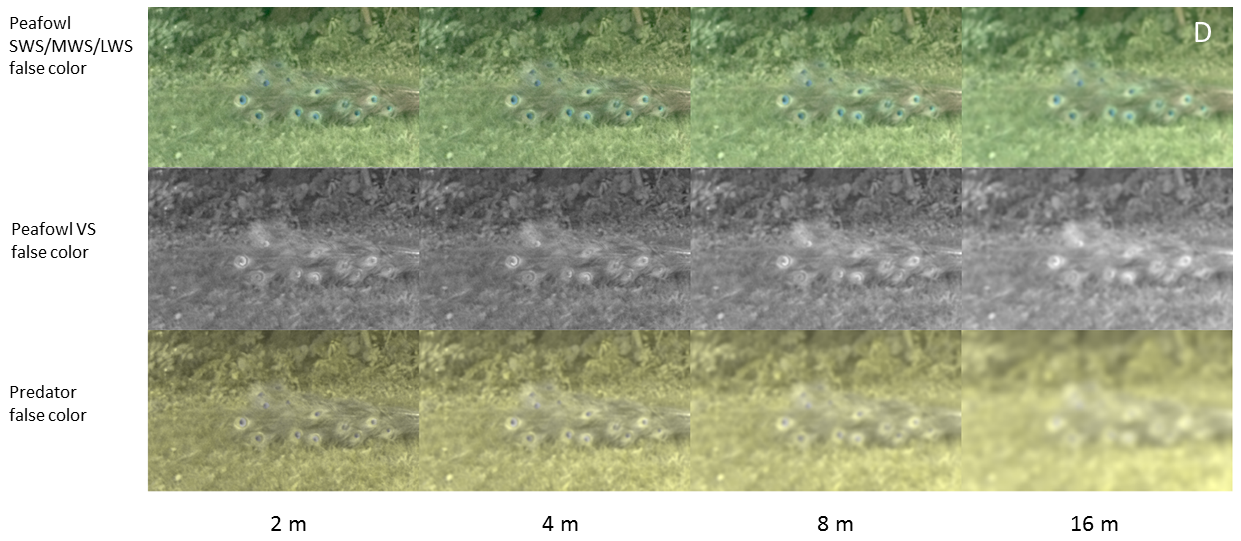
**

**S4 Fig D (caption on next page)**

**
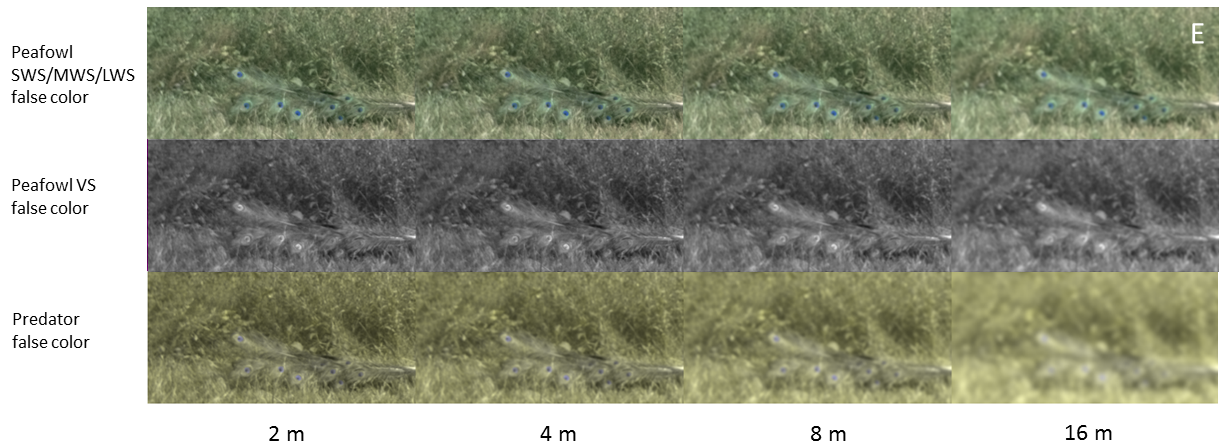
**

**S4 Fig. Model train false color images.** False color images for the peafowl and dichromatic mammalian predator visual systems, and predator luminance-only images, for four of the six model peacock train oriented (A-B) vertically and (C-E) horizontally.
