# Supplementary material for: How conspicuous are peacock eyespots and other colorful feathers in the eyes of mammalian predators?
Source: PLoS One. 2019 Apr 24;14(4):e0210924. doi: 10.1371/journal.pone.0210924 (PMC6481771; doi:10.1371/journal.pone.0210924)
Supplement: S5 Fig — (DOCX) [file pone.0210924.s009.docx]

**
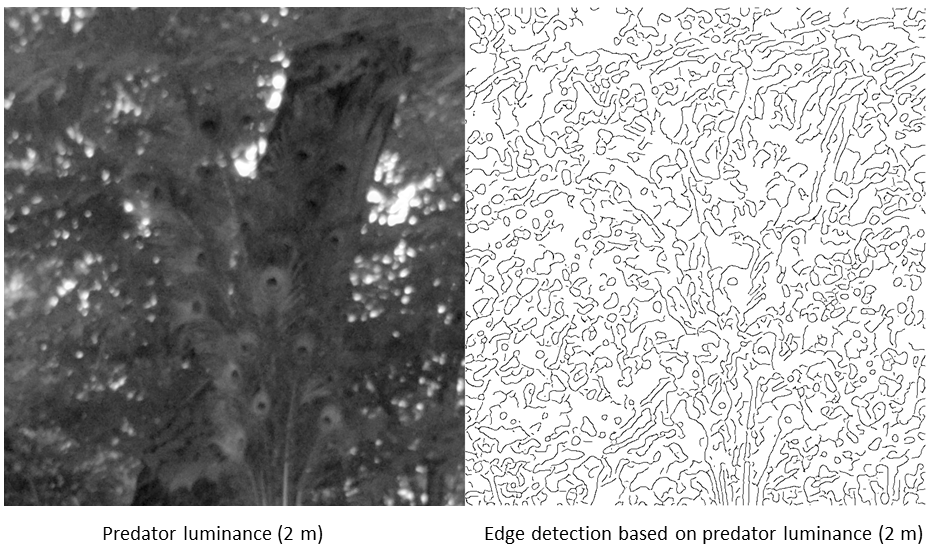
**

**A**

**
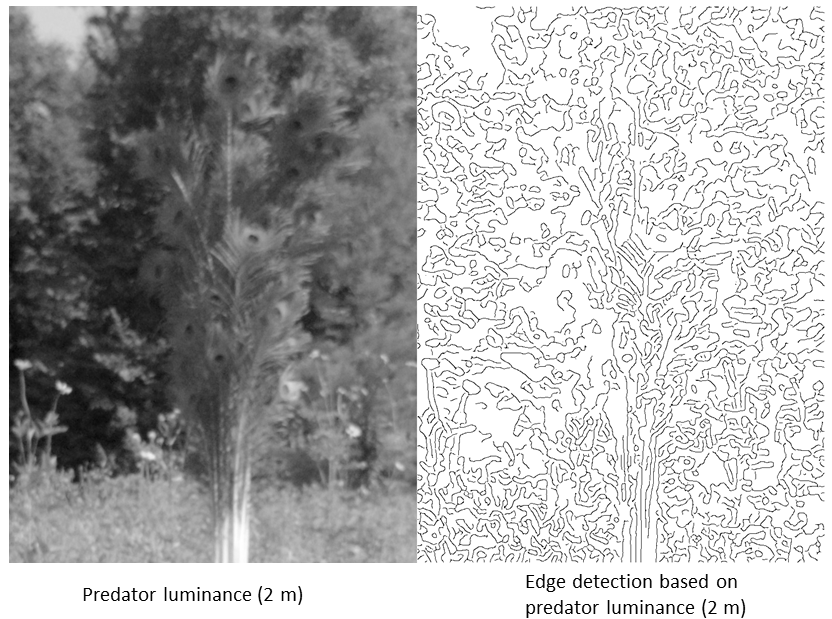
**

**B**

**S5 Fig (caption on next page)**

**
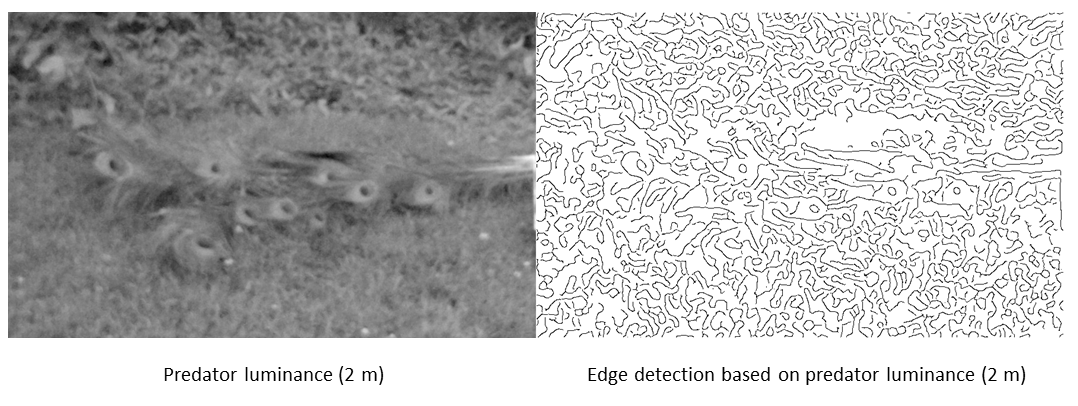
**

**C**

**
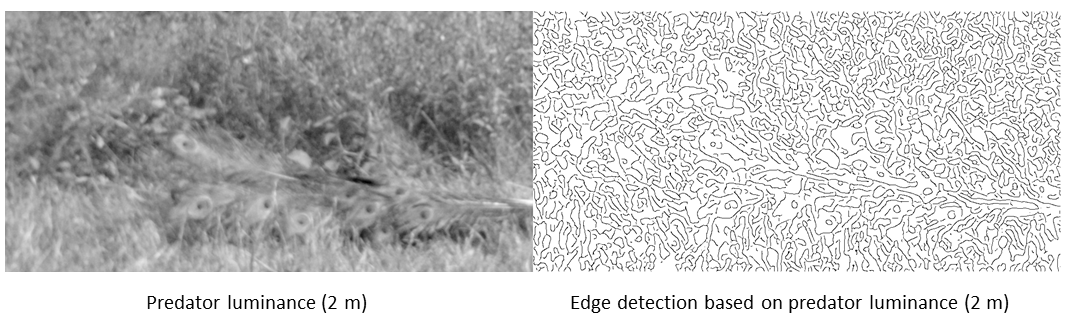
**

**D**

**
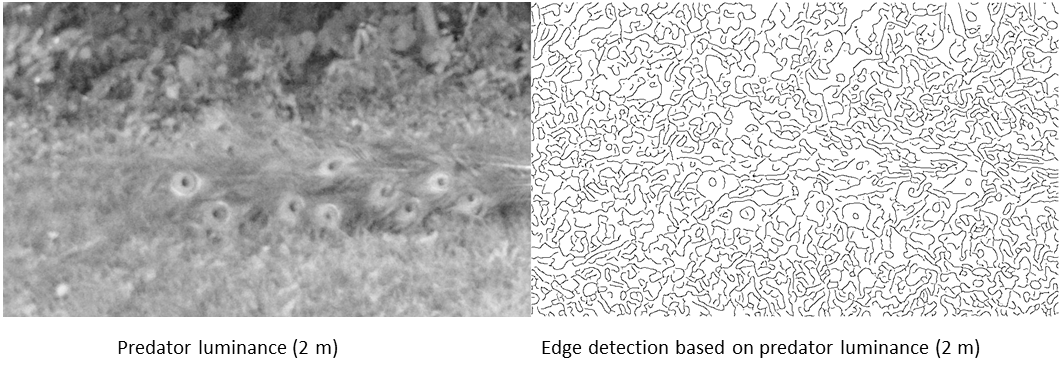
**

**E**

**S5 Fig. Model peacock train edge detected images.** Luminance-based images and edge detection images for dichromatic mammalian predator vision for the trains shown in S3 Fig. The model peacock train is oriented (A-B) vertically and (C-E) horizontally.
