# Supplementary material for: How conspicuous are peacock eyespots and other colorful feathers in the eyes of mammalian predators?
Source: PLoS One. 2019 Apr 24;14(4):e0210924. doi: 10.1371/journal.pone.0210924 (PMC6481771; doi:10.1371/journal.pone.0210924)
Supplement: S6 Fig — (DOCX) [file pone.0210924.s010.docx]

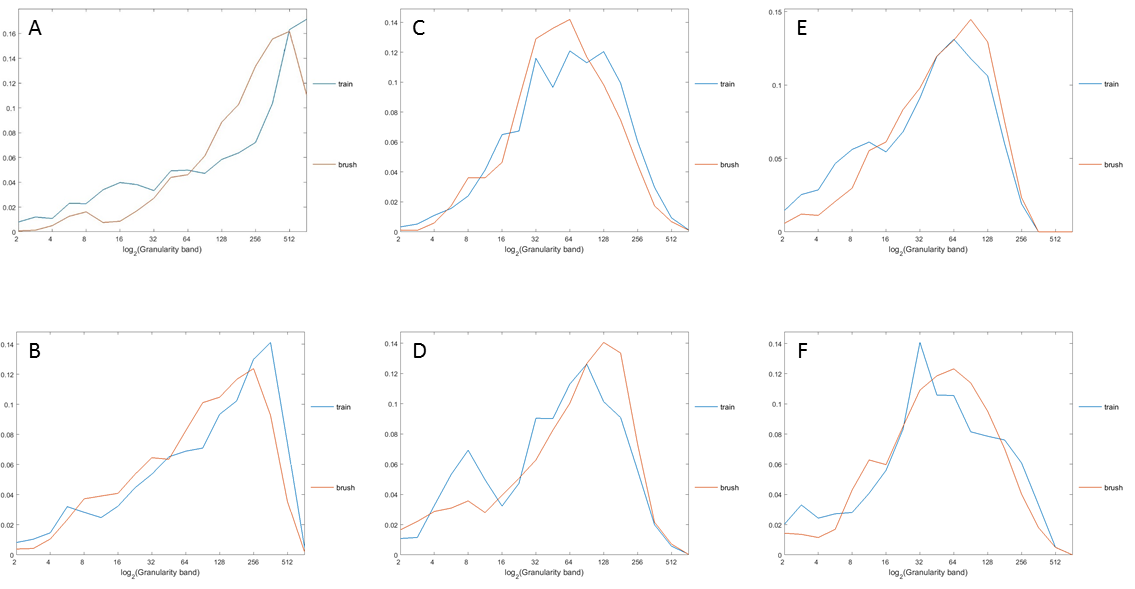


**S6 Fig (caption on next page)**


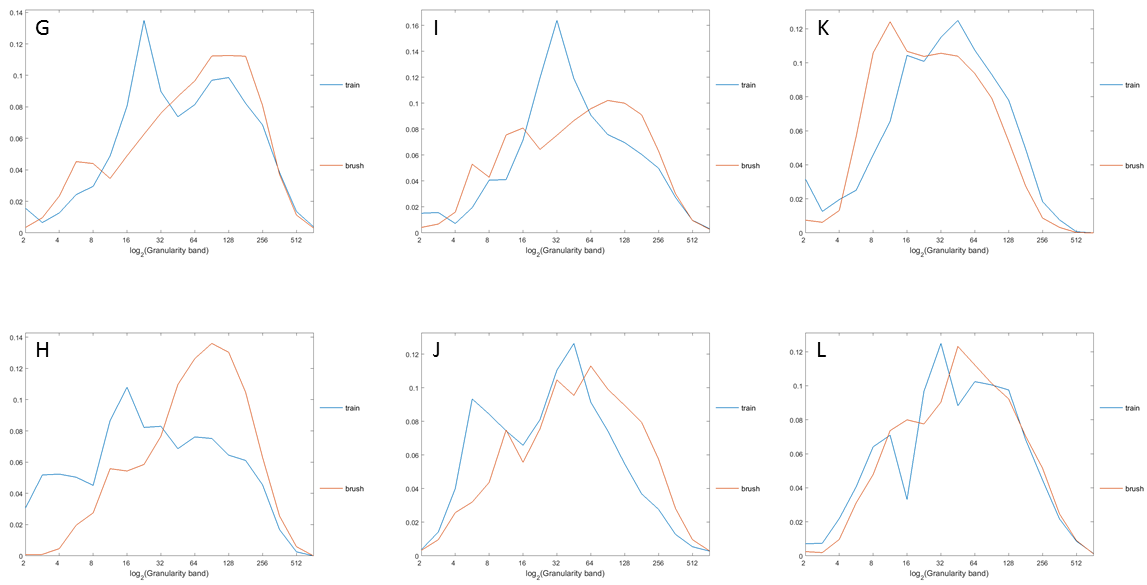


**S6 Fig (caption on next page)**


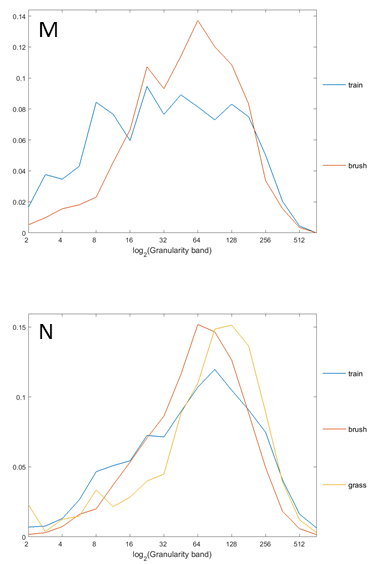


**S6 Fig. Pattern analysis of peacocks photographed against native foliage.** Granularity spectra computed for the trains of live peacocks and native background foliage using fourteen different photographs from online sources (S4 Appendix).
